# Water safety perceptions and practices across tourist sites in West Bengal, India

**DOI:** 10.3389/fpubh.2026.1821899

**Published:** 2026-08-17

**Authors:** Medhavi Gupta, Soumyadeep Bhaumik, Jagnoor Jagnoor

**Affiliations:** 1 George Institute for Global Health, New Delhi, India; 2 The George Institute for Global Health, Sydney, NSW, Australia

**Keywords:** behavior change, drowning prevention, injury prevention, tourist safety, water safety

## Abstract

**Objectives:**

Water-based tourism in India has grown rapidly, yet limited research has examined how tourists, operators, and local communities perceive water safety, implement protective practices, and navigate barriers to effective risk management. This study aimed to explore these perspectives across diverse tourist sites in West Bengal, a state with high drowning rates, to identify gaps and opportunities for strengthening injury prevention strategies.

**Methods:**

Seven water-related tourist sites representing rivers, beaches, and lakes were purposively selected across four districts of West Bengal. Qualitative in-depth interviews were conducted with tourists, tourism activity operators, and local residents until thematic saturation was reached. A total of 23 interviews were transcribed, translated, and analyzed using iterative thematic analysis to identify perceptions of risk, safety practices, and barriers and enablers to implementation.

**Results:**

Operators and locals perceived greater concern for water safety than tourists, largely due to lived experience and prior exposure to incidents. Risk perception was shaped by environmental conditions, seasonal changes, and cultural practices such as religious gatherings. Safety measures varied considerably across sites: urban and border areas demonstrated stronger enforcement of passenger limits and lifejacket use, while rural and religious sites often lacked adequate signage, surveillance, or infrastructure. Rescue response times were frequently delayed due to insufficient personnel and equipment, and alcohol consumption was consistently cited as a major risk factor. Participants emphasized the need for stronger regulation, improved crowd management during religious tourism, and greater public awareness of water hazards. Suggestions included building safe access platforms, expanding survival swim training, and enhancing police and operator rescue capacity.

**Conclusion:**

Findings highlight perceptions of inconsistent implementation of water safety practices across tourist sites in West Bengal, reflecting systemic gaps in regulation, enforcement, and infrastructure. As drowning risk is amplified by increasing tourism and climate change-related unpredictability, a proactive policy framework is urgently needed. Site-specific safety plans, standardized signage, improved rescue preparedness, and population-level sensitization were all suggested to reduce drowning risk and strengthen injury prevention. This study underscores the importance of embedding water safety within public health and tourism policy to protect vulnerable populations and ensure safer recreational environments.

## Background

Tourism in India has witnessed rapid growth over the past few decades, drawing millions of domestic and international visitors to its diverse landscapes, including to coastal, riverine and lake regions (1). Drowning is a major risk faced by tourists visiting recreational water bodies, and this population is often more likely to experience a drowning event than locals due to low awareness of risks, low familiarity with water environments, and riskier behavior (2, 3).

While research has focused on tourists in high-income contexts, few have surveyed water safety in tourism in low-and middle-income countries, exploring the safety measures currently implemented (4). Tourism locations may face different challenges such as lower prevalence of swimming skills in populations and poorer awareness on water risks in unknown contexts, issues that are exacerbated in low-and middle-income countries by the lower availability of fast and effective rescue services and poorer safety infrastructure (5, 6).

Eighteen percent of global drowning deaths are estimated to occur in India (6), where particularly high burdens are found in states with riverine, deltaic or coastal geographies. The state of West Bengal in eastern India is home to high-risk, deltaic environments but also experiences regular domestic tourist activity to water bodies, particularly along religious sites of the holy river Ganges. The region is also prone to frequent monsoon flooding and water exposure. A recent state survey in West Bengal found some of the highest drowning rates in the world, at 9.12 per 100,000 population, with a number of events being associated with alcohol consumption, recreational swimming and lack of safety infrastructure around water bodies (7). In tourism site contexts, factors such as limited public awareness, lack of trained lifeguards, insufficient signage, and disregard for local advisories may exacerbate the risks for tourists unfamiliar with local water conditions.

This study examines the experiences of water safety and risk perceptions of drowning across tourist sites in West Bengal and examines where implementation gaps may be occurring, impacting tourist safety. Addressing drowning in tourists will aid in the overall reduction of the high drowning burden in this state, and call attention to where further action is required.

## Methodology

This exploratory qualitative study applied a phenomenological approach (8) to explore perspectives on water safety for tourists in West Bengal, India. The qualitative approach allowed for the analysis of experiences toward water safety, current safe and unsafe tourism practices and barriers and enablers to these practices.

Study design was guided by the COREQ guidelines (Supplementary File 1). In-depth interviews (IDIs) were held with tourists, operators and locals in popular tourist spots along coasts or rivers in West Bengal. Seven sites were chosen to cover a range of waterbodies (riverine, beach and lake), and these were spread across the following districts: Purba Medinipur, Kolkata, North 24 Parganas and Murshidabad. This provided a variety of rural, semi-urban and urban tourist sites.

### Participants

Eligible participants were adults able to give informed consent who were involved in tourism, either as a tourist, a tourism activity operator or other local with exposure to tourists. Participant selection was aided by a local NGO partner (Child in Need Institute) engaged for a larger project on drowning in West Bengal with strong ties to communities across the state. Participants were approached at the sites purposively (9) to ensure a mix of these three types of individuals. The NGO representative assisted the data collectors in navigating tourist sites, such as advising on appropriate times to visit, and identifying who to approach based on their familiarity with who was likely to be a tourist, an operator or a local resident. Participants were approached by the data collector and note taking pair from the research team, who explained the study and provided participants with a Participant Information Statement. Participants were then interviewed in a quiet and private area of the tourist site at the time of recruitment.

### Data collection

Data was collected by three trained data collectors hired for the project (two female and one male), all with at least a master’s degree in a relevant field and previous experience in qualitative data collection. Each interview was conducted by two data collectors–one interviewed the participant while the other took notes on key responses and ensured the interview guide was followed, while also asking follow up questions where appropriate. All interviews were held face-to-face and audio recorded. To ensure comfort of participants, all data collectors were fluent Bengali speakers from the state. The interviews were later transcribed and translated by a trained member of the research team fluent in Bengali.

In-depth interviews were guided by a semi-structured tool which allowed the interviewer to ask probing questions (Supplementary File 2). The interview guide was pilot tested with two potential participants at a tourist site not included in the study. Some additional probing questions were added to better aid the data collector after the pilot testing was completed. Interviews were conducted in Bengali, the local language, in a private location near the tourist site with only interviewer and participant present. After each interview, key points as noted by the note-taker and reviewed by the interviewer were shared with the research team, including author SB, to assess for saturation. Transcripts were not returned to participants.

To reduce the influence of these prior perspectives of the researchers, interviewers followed a semi-structured guide, encouraged participants to share both positive and negative experiences, and avoided leading questions. The interviewers had no prior relationship with participants, and no incentives were provided for participation.

### Data analysis

As the interviews progressed, themes were discussed iteratively. At the end of every day of data collection we conducted debrief as a team using a structured format which also included field notes. The structured format asked the data collector to note 5–10 key points of the interview and separately listed any new points for the participant type to allow for the tracking of saturation across the whole study sample. No new changes were made to the interview guide as data collection continued, as themes found were relevant to questions already in the guide, but the data collectors were sometimes provided feedback on which points to probe for further detail. Data collection was stopped after at least two IDIs were conducted at each of the seven sites and data saturation was reached for each type of participant (tour operator, tourist and local).

Iterative thematic analysis guided the coding of the interview transcript content, using an inductive thematic approach after data collection was completed (10). Through repeated review of the data for familiarization, codes were deductively grouped into broader categories relating to perceptions of risk, safety practices, and barriers and enablers to implementation, from which themes and sub-themes were inductively developed and refined. One researcher (MG) coded the transcripts against perceptions, safety practices, and enablers and barriers to safety practices in NVivo 11 software. Sub-themes under each of these headings were then compiled. These were reviewed and triangulated by the other authors and the data collection supervisor against notes taken during the interviews and debrief notes. Where authors and the supervisor did not agree, the transcripts were re-examined and re-discussed, and consensus built. This allowed for all authors to challenge assumptions and ensure findings remained grounded in the data.

### Ethics

All participants provided written informed consent. Participants were informed that participation was confidential and explained the study aims and how data would be used. They were assured that identifiable data would not be maintained in the transcriptions. Participants were also made aware of the data collectors’ role in the project. Ethical approval was granted The George Institute for Global Health (India) Ethics Committee in New Delhi, India (03/2024), the Institutional Ethics Committee of Institute of Health and Family Welfare, Government of West Bengal, India (IHFW/IEC/7389) and Health Ministry’s Screening Committee (HMSC), Department of Health and Family Welfare, Government of India (202417898).

## Results

A total of 23 interviews were held, spread across seven tourist sites. No repeat interviews were carried out, and no participants refused participation after approach. Six participants were tourists, nine participants were tourist activity operators, and eight participants were other locals living near the tourist site with regular interaction with tourists. All participants were local Indians, as these locations are rarely frequented by international visitors. Four participants were women. Interviews lasted between 10 to 30 min. Thematic analysis of the interviews identified three key themes, as discussed below, and illustrative quotations are also provided.

Table 1 summarizes the key themes and sub-themes identified, which are then explained in greater detail further in the results section.

**Table 1:** Key themes and sub-themes.

Primary theme	Sub-theme
Theme 1: Risk perception is shaped by environment, experience, and belief systems	Risk perception was mediated by type of environment, such as lake or river, and signage should be tailored to ensure awareness of risks Risk was considered high at sites of religious tourism Operators and locals expressed greater awareness of water safety issues than tourists Participants believed safety was both an individual and government responsibility Alcohol was a significant risk factor identified
Theme 2: Patchy implementation of water safety measures across the state	Implementation and adherence to safety measures varied significantly across sites; urban sites showed better adherence Boat and boat operator licensing and training was standardized, lifejacket wear and passenger limits were well-followed Though safety infrastructure was present at religious sites, it was often unmaintained Rescue services are under-resourced and insufficient Presence of warning signage is inconsistent There is limited enforcement of safety rules
Theme 3: Perceptions toward improving safety practices	Participants identified overall positive trends in safety practice Positive changes to safety practices were reactionary Strengthening rescue capacity and responder training is viewed as a priority Improved infrastructure is needed to support safe access to water during religious rites Climate change is increasing the difficulty of implementation of safety practices Population-level water safety education and survival swimming skills are needed.

### Theme 1: risk perception is shaped by environment, experience, and belief systems

Participants’ perspectives of water safety risk were mediated through an interplay of environment, experience, and belief systems. Where beaches were prone to high waves and changing tides, rivers presented strong currents that made swimming difficult. In lake areas, unexpected deep water was considered a challenge. For lakes and rivers, monsoon season was considered a risky time as the waterbodies’ size would increase. Participants noted the need for specified warnings and signages to warn potential swimmers of risks and danger zones.

*“During the rainy season, when pits form, the current becomes strong. People often don’t realize it. They step into what looks like shallow water but suddenly get pulled into deeper currents.” – Purba Medinipur IDI21, Tourist operator*

Risk was also considered high in sites that were popular for religious tourism, as these would attract large crowds during festivals and auspicious days. While some locals interviewed in the organisation of religious events noted attention to crowd control and surveillance from religious institutions and police, these actions were not considered adequate for the number of attendees.

*“In Shravan, many people come to pour water at the Shiva temple. During this time, some incidents have happened.” – Murshidabad IDI17, Local*

Risk perception also differed by type of respondent. Operators and locals expressed greater awareness and concern for water safety than tourists, as many had witnessed incidents at their sites or had been sensitized during operator training. Many tourists were not aware of water safety practices or how to identify safe or dangerous swimming locations but were confident in their abilities even if they had not received survival swim training.

*“We taught ourselves [to swim] …we have learned ourselves … you can then use that skill to save people if there is an accident.” – Kolkata IDI5, Tourist*

However, despite differences across types of water bodies, two common attitudes were identified. Firstly, participants noted that water safety was an individual responsibility in addition to being a government responsibility, and that tourists’ reckless behavior was concerning. Parents were also blamed for not supervising children sufficiently.

*“Ultimately, people must be responsible for themselves. If civic police or we spot them, we will warn them not to go too far.” – Purba Medinipore IDI22, Tourist operator*

*“Yes, especially when they go boating, they are told to wear life jackets. But sometimes they don’t listen to us. Once they get into the water, they act differently, and we can’t do much.” – North 24 Parganas IDI8, Tourist operator*

Secondly, alcohol was identified as a significant risk factor across many sites, as tourists sometimes entered the water or boarded boats inebriated. While boat operators tried to prevent inebriated passengers from boarding, they were unable to prevent all from entering the water themselves unless police were available to intervene.

*“Interviewer: Why do you think such accidents happen?*

*Participant: Mainly because of alcohol. People don’t listen to the civic police and go swimming far into the sea, where strong currents pull them away.” – Purba Medinipore IDI22, Tourist operator*

Lastly, while many participants noted individual responsibility for their own safety, they also acknowledged that individuals would be unable to navigate risk if they were not aware of hazards. At sites where safety practices were well-implemented and regulated, the key barrier to safety was tourists’ own awareness of how to navigate the specific water body.

*“There are rules, but they’re not always clearly displayed.” – Kolkata IDI1, Tourist operator*

*“Presently, arrangements are okay. Tourists are somewhat safe due to BSF’s [Border Security Force’s] strict monitoring, but more awareness programs could help.” – 24 North Parganas IDI12, Local*

### Theme 2: patchy implementation of water safety measures across the state

Overall, implementation and adherence to safety measures varied significantly across sites. At some sites, especially urban or border areas, participants noted an improvement over the past decade in the availability of patrolling civic police or border security forces, (who enforced regulations such as passenger limits), life jacket usage, and limitations on boating activities at night. There were also provisions to close tourist sites at the time of a storm, cyclone, or high currents.

*“Yes, it’s a good change … sometimes 50 people would board a boat, which was highly unsafe. Now only 10 passengers are allowed, and each must wear a life jacket. This is much safer.” – North 24 Pargana IDI12, Local*

*“Yes, civic police are deployed here. They patrol every half or one hour, especially when many people are bathing.” – Purba Medinipore IDI22, Tourist operator*

Furthermore, at most sites, boat operators were required to be licensed and undergo accredited training on safe rescue and resuscitation. Training was refreshed every few years, and tour companies enforced training requirements on their employees.

*“Yes, after receiving training there, they give us a driving license, and only then are [the tourist operators] allowed to operate.” – Purba Medinipore IDI21, Tourist operator*

*“Yes, I’ve taken disaster management training. If someone swallows water, I lay them down and apply pressure to their stomach to help expel the water. If unconscious, I perform mouth-to-mouth resuscitation.” – North 24 Parganas IDI8, Tourist operator*

At some sites, particularly religious sites where crowds can increase dramatically during auspicious events, authorities placed barriers to prevent tourists from entering unsafe or deep water and installed CCTV cameras. Infrastructure was also in place in some river areas to control flood waters. However, some tourist operators and locals noted that the surveillance infrastructure was not regularly maintained, and they were uncertain if it was functional.

*“On such occasions, temporary barriers are placed to restrict people from entering deep into the river.” – North 24 Parganas IDI16, Local*

*“We need more surveillance. Some cameras are installed, but I’m not sure they’re all working. That footage could be useful during incidents.” – Kolkata IDI1, Tourist operator*

While some regulations such as passenger limits and life jacket wear are well-followed, a significant challenge still occurred at the time of rescue and resuscitation. Rescues still often took at least 15 min if not much more, as the boat operators first called for a nearby operator or civic police, who then entered the water. Inconsistent availability of responders such as marine police personnel also hindered rescue.

*“Interviewer: Is there an instant rescue system in place?*

*Respondent: No. We must call the police, who arrange for divers to search. No immediate rescue services are available.” – North 24 Parganas IDI13, Local*

The presence of signage and warnings was also patchy, with many sites displaying minimal risk-related communication. While some sites used loudspeaker warning systems, few had any written or infographic warnings.

*“We use loudspeakers to make announcements.” – North 24 Parganas IDI8, Tourist operator*

*“You can tell by watching the water rise, but there’s no outside help or warning—you have to figure it out yourself.” – Murshidabad IDI20, Tourist*

However, at some sites, while tourist operators and even locals were aware of regulations, they noted there was no enforcement and operators and tourists did not follow rules. There were also no police or rescue services available. These sites garnered less civic attention than those near larger populations or near security-related inflection points, forcing operators to often managed rescues themselves.

*“All this has not been done according to the guidelines - there are some boatmen who do not limit the boarding of passengers... they exceed the capacity of 12 to 14 people.” – North 24 Parganas IDI7, Local*

*“We [operators] take the boat, throw life jackets, pull them up, and bring them back. If jackets slip off, we jump in ourselves.” – Purba Medinipur IDI22, Tourist operator*

### Theme 3: perceptions toward improving safety practices

Participants overall demonstrated positive perceptions toward improving safety practices in tourism, but these would be met with challenges.

While participants cited improvements in regulation, these changes were usually brought about by previous fatal drowning incidents, suggesting a reactive approach. These measures were sometimes restrictive rather than preventative, such as shutting down access to water entirely.

*“It happened either one or three years ago. Someone went to bathe and drowned. There was panic. Since then, there's been a ban. If the ghat had been built properly, maybe it could’ve been avoided.” – Murshidabad IDI17, Local*

While the presence of civic police can be improved, many police do not receive appropriate search and rescue training or are unable to swim. This would decrease their capability to conduct appropriate rescue.

*“This is one thing that the (Border Security Forces) BSF personnel on duty here should be well trained here and know how to do duty in the river because as far as I have heard, the person who was on duty there did not know proper swimming.” – North 24 Parganas IDI7, Local*

Another key deficient intervention across most sites was infrastructure to support safe access to water, particularly for people with disabilities and the older adults at religious sites. Most sites did not have ramps, stairs, or paved areas for people to access water safely.

*“Some arrangements are still needed. Especially for the older adults and disabled, more facilities should be provided. There should be proper management at the skywalk, at the ghats, and for the tourists.” – North 24 Parganas IDI15, Local*

Climate change-related erosion and dangerous weather were also cited as barriers to implementing safety practices. Participants, especially operators and locals, noted an increase of flooding events, and greater unpredictability of rivers and coastal currents, increasing the danger to tourists. This made early warnings more difficult, and complicated rescue efforts.

*“The river here is unpredictable. Once, around 8:00 in the evening, a storm came suddenly. Border Security Force (BSF) members were also swept away.” – North 24 Parganas IDI7, Tourist operator*

Some tourists noted that wider population training, particularly survival swim training, was essential to improving water safety, as site-specific safety practices were not enough to prevent all mishaps. The general population was not aware of water risks and how to manage them.

*“Parents suffer from so much phobia with water that they do not want to enrol their child in swimming.” – Kolkata IDI4, Local*

*“Government should implement swimming space for every school that should be obviously free of cost… it should be a government initiative.” – Kolkata IDI4, Local*

## Discussion

The current study aimed to analyze community perceptions toward the water safety situation for tourists in an LMIC context, specifically the state of West Bengal in India. First, we found that perceptions of drowning risk were shaped by environmental conditions, prior experiences, and individual belief systems, with operators and local residents generally demonstrated higher risk awareness than tourists. Second, the implementation of water safety measures was perceived to be inconsistent across sites, with substantial variation in the availability and enforcement of regulations, safety infrastructure, signage, and rescue services. Third, participants expressed strong support for improving water safety but highlighted the need for a more proactive approach, including strengthened rescue capacity, safer infrastructure, greater public awareness, and adaptation to emerging challenges such as climate change and increasing tourist numbers.

The study found varied experiences relating to water safety, including differing risk profiles across different types of tourist sites. These findings demonstrate that water safety practices may need to be tailored to site-specific conditions to maximize effectiveness. Currently, while there are some localized actions by marine police and border security forces to manage water safety, this is *ad-hoc* and reactive to previous incidents. This suggests that safety planning is not a priority at a systems level. While state and national policy can provide guidance on safety practices, the variable risk factors across different types of sites - lakes, rivers and beaches - speak to the need for comprehensive site-specific assessments of vulnerabilities, risks, and hazards for drowning to guide the development of specific safety plans for sites. Proactive safety planning for these sites should be tailored to evolving tourism patterns, such as increases in religious tourism or to sites made popular by social media (11, 12). For this, data collection on tourist visits can enable the prediction of high-risk situations, such as around festivals and school holidays, where patrolling and rescue resources should be increased. Using state and national guidelines as a starting point, local governments can develop and implement context-specific plans to mitigate risk at the tourist sites in their respective regions.

The first theme also identified poor management of religious tourism around water bodies, highlighting the need for improved planning and resourcing for crowd control during religious events at water bodies, such as recent reports of drownings during *Jivitputrika Vrat* celebrations which are held along the Ganges river. Such tragedies often occur in marginalized communities who are not aware of risks and may not have access to disaster or flood communications, as well as social pressure to participate even in high-risk situations (13). India does not have a specific regulatory framework for the management of large-scale religious events (14). Predictive data on attendance to develop plans, ticketing systems, the provision of trained and resourced rescue services, appropriate signage and water-entry infrastructure are all essential (15).

The second theme found patchy implementation of water safety measures across the state, reflective of non-standardized safety practice. The West Bengal tourism policy contains limited references to water-related activities, with some mention of boats requiring maximum passenger limits. There is no discussion of rescue training for marine police or emergency responders, use of lifejackets, warnings, signage, or water body-specific requirements for activities and access (16). The first step to reliable water safety practices is for state and national policy to define requirements and embed those within tourism policy to ensure accountability and tracking. Embedment in policy will also release resources to support safety practices, such as ensuring enough emergency equipment and personnel for responsive rescue, early warning systems, and patrolling (1, 17). Water safety as a concept must be considered more crucial by policymakers and the public through campaigns and stronger messaging (7).

The third theme provided insights into how specific safety practices may be improved. The lack of signage, warnings and overall awareness of water safety was demonstrated through lower risk perception in tourists versus operators and locals. Many sites did not have signage to designate safe swimming zones or provide warnings, though some sites did broadcast loudspeaker messages. The attitude of tourists suggests that these efforts are not enough. Signage and warnings may be increased and should be tailored to the environment type, such as to warn of deep water and presence of dangerous currents in different parts of the water body. Furthermore, these signs should follow a state or national standardized appearance so they can be immediately recognizable across sites and become part of the public conscience (18). Interestingly, risk perceptions of locals and tourist operators and tourists themselves contrasted, where tourists were often less aware of safety risks, and perhaps less concerned. This lends to the conclusion that individual water safety behavior must be addressed at a population level, such as by embedding water safety into school education to improve awareness of possible risks when entering waterbodies (6). This study also identified that building and maintenance of safety infrastructure, provision of trained lifeguards, and enforcement of boating regulations could bridge implementation gaps across all sites. These are in line with WHO recommendations (6).

## Limitations

The current study conducted a range of qualitative interviews across a variety of tourist sites to provide nuanced understanding of the state of tourist water safety in the state of West Bengal in India. However, while this study visited seven tourist sites in West Bengal across four districts, the findings may not be generalizable to all tourist sites in the state or across the country, which would require a larger sample size across multiple states. Additionally, interviews with civic police or border security personnel may have been insightful from a systems perspective, but those required government permissions. Future work should also consider understanding policymakers and government perceptions toward tourist water safety to identify how policy change may be brought about.

Though data from tourists appeared to reach saturation after six participants, further data may have enriched the findings. In addition, while team discussions were conducted throughout data collection to enhance credibility of the findings, the data was not re-checked by participants, which may have introduced researcher bias. In addition, recruitment of women proved challenging due to the male-dominated nature of work in these areas, but future research should consider women’s perspectives and experiences and how these differ from men’s. Future research should also consider more objective measures of ground realities, as qualitative perceptions may provide a biased or incomplete explanation of safety practices.

Lastly, due to time and resource constraints, only one author conducted coding. Though key findings, themes and sub-themes were assessed against field notes and debrief notes and discussed with all authors and the data collection supervisor, double coding may have strengthened the analysis.

## Conclusion

This study aimed to develop an understanding of perceptions toward water safety practices at tourist sites in West Bengal, India. This study found that perceptions and experiences of water safety practices were shaped by environmental conditions, lived experience, and individual beliefs. Participants also described inconsistent implementation of safety measures across tourist sites and highlighted the need for more proactive, system-wide approaches to water safety, including stronger regulation, improved infrastructure and rescue capacity, and greater public awareness and education. Standardized processes to identify and implement site-specific safety plans are required at state and national level to mobilize resources and create incentives for consistent application of interventions such as passenger limits, fast rescue and lifejacket wear. Individual safety behavior can also be improved through population-level sensitization and better signage and warnings.

## Data Availability

The raw data supporting the conclusions of this article will be made available by the authors, without undue reservation.
