# Supplementary material for: Water safety perceptions and practices across tourist sites in West Bengal, India
Source: Front Public Health. 2026 Aug 17;14:1821899. doi: 10.3389/fpubh.2026.1821899 (PMC13526560; doi:10.3389/fpubh.2026.1821899)
Supplement: Supplementary file 2 [file Table_2.DOCX]

**Supplementary File 2 - Topic guide for IDI**

**Interview Details**

Participant ID: ………... /…. ( District Name / IDI Starting from 01,02, 03…)

Location of interview:

Researcher Initials |__|__|__|

Note taker initials |__|__|__|

Date |__|__/__|__/__|__|

**Introduction**

Thank you for agreeing to give the interview. I appreciate your willingness to participate and the time you have taken out of your day to take part. I am ______________________________ from ______________________ and the ___________________, my colleague is the note-taker. We are conducting these interviews in to understand your views and experiences in relation to drowning in tourists and related water safety in West Bengal, India. We would like to find out from you what your thoughts/opinions and concerns are about drowning and water safety. This will help us address drowning in the state.

- Explain consent.
- Give opportunities to ask for clarification.
- Ensure written informed consent is taken and participant information sheet copy is given to participant.

**Participant Background**

Now I would like to know more about you. Can you please tell us your name, age, occupation.

1. Gender of participant (circle as appropriate)
   - Male
   - Female
   - Others
2. Age of participant (in years):
3. Occupation details
4. Participant category

- Tourists visiting a sea-beach or a tourist spot with water body.
- Those related to the tourism industry in sea-beach or a tourist spot with water body.
- Lifeguards in sea-beach or a tourist spot with water body.

**Topic Guide**

| **Domain** | **Topics** |
| --- | --- |
| **Perceptions of drowning risk and water safety** | - **What concerns do you have about safety of tourists in this spot?**   **Probe**   - timing of access - safety around tourist spot - crowding - **What about drowning? Do you think there is a risk of drowning in this tourist spot? Do you think water safety here is adequate?**   **Probe**   - Use of life jackets / floating buoys - presence of safety measures - presence of lifeguards |
| **Safe and unsafe practices** | - **What are some of the reasons you think there is compromised in safety here?**   **Probe**   - Knowledge or attitude of tourists - Staff presence - Situations when risk is higher (alcoholism) - Selfies-social media - Perceptions on who is more vulnerable and why. - **How do you ensure safety of tourists? (do not ask to tourists)**   **Probe**   - Concerns around weather conditions - Concerns around Infrastructure / signboards - Concerns around rescue and resuscitation - **How do you ensure your personal safety as a tourist?**   **(Only for tourists)**  **Probe**   - Information seeking - Signboards - Special precautions - Timings - Selfies-social media - Activity restriction - **What are the enablers and barriers to accessing safety of tourists?**   **Probe**   - Government support / policy - Distinctives and incentives around tourist water safety |
|  |  |
|  |  |
| **Experience of drowning/ water safety compromise events** | - **Have you heard of or had any incident (in this or any other tourist spot in West Bengal, India?**   **Please describe the situation. What happened? How and when did this happen?**  Probe   - Any rescue - Pulling of water - Government action on safety - **Do you know what the usual immediate practices are after a person has recovered from water? (Not for tourists)**   **Probe**   - Equipment - Training - Confidence - Support |
|  |  |
|  |  |
| **Suggestions for improvement** | - **What can be done to improve safety / reduce drowning related to tourists?** |
| **Exit question** | Do you have anything else would like to say or mention further, which you think will be useful for us to know |
| **Thank you** | |
